# Neuroimaging in Dementia With Lewy Bodies

**DOI:** 10.7759/cureus.15694

**Published:** 2021-06-16

**Authors:** Abhishikta Saha, Dipanjan Banerjee

**Affiliations:** 1 General Medicine, Pennine Acute Hospitals NHS Trust, Manchester, GBR; 2 Internal Medicine, East Sussex Healthcare NHS Trust, Hastings, GBR

**Keywords:** dementia with lewy bodies, neuroimaging, spect, fdg, mibg

## Abstract

Dementia with Lewy bodies (DLB) is one of the most common forms of dementia. It can present as neurocognitive decline, visual hallucinations, and concomitant symptoms of rapid eye movement (REM) sleep behavior disorder. Early diagnosis remains one of the cornerstones of managing this form of neurocognitive disorder but, often, making an early and accurate diagnosis can prove to be challenging. For this article, our goal was to review the utility of various neuroimaging modalities in making a swift and accurate diagnosis of DLB. We used PubMed to look for helpful, informative, and peer-reviewed articles. We discussed the role of a plethora of different imaging techniques, ranging from structural imaging like computed tomography (CT) and magnetic resonance imaging (MRI) to molecular imaging (single-photon emission computed tomography, positron emission to- tomography) as a diagnostic tool for DLB. We arrived at the conclusion that these novel neuroimaging modalities have already proven to be very helpful in ruling out differentials and making an early diagnosis of DLB. However, ongoing research is required to increase the diagnostic accuracy, leading to the early identification and treatment of DLB.

## Introduction and background

"The disease might hide the person underneath, but there is still a person underneath who needs your love and attention" - Jamie Calandriello.

There are almost around 850,000 people with dementia in the United Kingdom. The number is thought to rise to 1.6 million by 2040; every one in six people over 80 will have dementia [1]. Dementia with Lewy bodies (DLB) is one of the most common causes of dementia or neurodegenerative disorders, only second to Alzheimer’s disease (AD) [2]. There are no curative treatments available for DLB yet. However, doctors and scientists across the globe agree that once such treatments are available, patients would most likely benefit from the initiation of treatment as early as possible, which makes early diagnosis of DLB a crucial factor [3]. Diagnosis of DLB remains challenging, and statistics reveal that only 33% of cases are correctly diagnosed [4]. Hence, the most crucial hurdle to overcome in the management of DLB lies in making an early diagnosis and accurately ruling out other possible differentials [5]. Biomarkers are defined as substances in the body that can be objectively measured and able to signify an underlying disease process, preferably at an early stage of the disease [6]. Neuroimaging plays an integral role in the identification of these biomarkers and in this article, we aim to provide a brief review of imaging modalities contributing towards early diagnosis of DLB.

## Review

Method

We have used PubMed as the search engine for this literature review. As this is not a systematic review, we have not followed PRISMA (Preferred Reporting Items for Systematic Reviews and Meta-Analyses). We have only considered studies performed within the past 10 years specifically on humans. Citations, year of study, subject population were taken into consideration when selecting studies to review from. Studies done on other species or older were not excluded. A total of 33 peer-reviewed articles were included in this study. Data collection has been done ethically and legally to the best of our knowledge.

Discussion

Dementia With Lewy Bodies and Its Aetiologies

Lewy Body Dementia/Disease (LBD), otherwise known as dementia with Lewy bodies (DLB), is a neurodegenerative disorder due to pathologic deposits of alpha-synuclein in the brain [7]. DLB presents with an insidious onset and slow progression of symptoms, classified into core and suggestive features. The core features of DLB include varying levels of cognition with remarkable fluctuations in attention and alertness, recurrent visual hallucinations, Parkinsonism-like features [7]. The other suggestive features of DLB consist of similar symptoms indicative of rapid eye movement sleep behavior disorder and severe neuroleptic sensitivity. Clinically, DLB can be notoriously difficult to distinguish from Parkinson’s disease dementia (PDD), where the motor symptoms usually precede the appearance of cognitive symptoms. DLB can also co-exist with other etiologies of dementia, including AD, especially in the older age group [7].

Structural Imaging

Structural imaging techniques, such as magnetic resonance imaging MRI) and computed tomography (CT), are very useful in performing volumetric studies in DLB [8]. Structural studies are often also used in the clinical setting to rule out differential diagnoses of various etiologies of dementia, including cerebrovascular disease or intracerebral space-occupying lesions [8]. Using cortical thickness measurement in MRI, Barber et al. found that DLB patients had less volume loss in the regions of the temporal lobe, amygdala, and hippocampus than AD, and no such volumetric difference was observed when compared to vascular dementia (VaD) [9]. They also observed an excellent correlation between periventricular hyperintensities and ventricular dilation with increasing age [9]. The grey matter loss in the temporal lobe is also found to be less pronounced in DLB than in AD [10]. Using voxel-based morphometry, Burton et al. found that while extensive grey matter loss in the bilateral occipital lobe, temporal lobe, frontal lobe, and left parietal lobe seems to be the dominant finding in PDD, and, PD without dementia was found to be associated with only frontal lobe atrophy [11]. However, no such distinguishable features were found between PDD and DLB [11]. A study by Sanchez-Castaneda et al., which was carried out on 12 clinically diagnosed DLB and 15 PDD patients demonstrated that visual symptoms correlated well with more grey matter loss, which was significantly pronounced in the right inferior frontal gyrus in DLB patients and left orbitofrontal lobe in PDD patients [12]. Using the Sparse Partial Least Squares (SPLS) classification of cortical thickness measurements on a total of 97 subjects with AD and DLB, Lebedev et al. demonstrated different patterns of atrophy between AD and DLB with 82.1% sensitivity and 85.7% specificity [13]. Hence, the differential diagnosis can be aided by analyzing the unique patterns of cortical volume loss observed in different etiologies of neurodegenerative disorders [13]. The findings of several relevant structural studies have been summarized in Table 1.

**Table 1:** Summary of findings in structural imaging HV: healthy variants; AD: Alzheimer's disease; DLB: Dementia with Lewy bodies; PDD: Parkinson's disease with dementia; MRI: magnetic resonance imaging

Author	Study performed	Subjects	Findings
Barber et al.	T(1)-weighted, T(2)-weighted, and proton density MRI	25 AD and 27 DLB	AD is characterized by more pronounced atrophy in the temporal lobe, amygdala, and hippocampus, which is relatively spared in DLB. Periventricular hyperintensities correlate well with age while deep white matter hyperintensities correlate well with a history of hypertension [9].
Ballmaier et al.	MRI	29 AD, 16 DLB, 38 HV	More pronounced grey matter atrophy in orbitofrontal and temporal regions in AD compared to that of DLB [10].
Burton et al.	Voxel-based morphometry	26 PDD, 31 PD, 28 AD, 17 DLB, 36 HV	Extensive volume loss in PDD involving the frontal lobe, temporal lobe, including the hippocampal and parahippocampal gyrus, occipital lobe, while PD with dementia showed such changes only confined to the frontal lobe. No significant differences could be observed between PDD and DLB [11].
Sanchez- Castaneda et al.	Voxel-based morphometry	12 DLB and 15 PDD	Visual symptoms are increasingly found to be associated with a higher amount of grey matter loss. In DLB, this is found to be focused around the right inferior frontal gyrus while in PDD it is more pronounced around the region of the left orbitofrontal lobe [12].
Lebedev et al.	Sparse partial least squares (SPLS) classification of cortical thickness measurements in MRI	Two cohorts involving 97 AD and DLB subjects	Subjects with AD showed atrophy in the mid-anterior temporal, occipital, and subgenual cingulate cortex while DLB was characterized by a unique pattern of cortical thinning in the regions of dorsal cingulate, posterior temporal, and lateral orbitofrontal area [13].

Metabolic Studies

Molecular imaging modalities, such as single-photon emission tomography (SPECT) and fluorine-18-fluorodeoxyglucose positron emission imaging (FDG-PET), have played a pivotal role in understanding the complex pathophysiology of DLB by assessing metabolic activity in vivo with the correct radiotracers involved [14]. In DLB, metabolic studies show hypometabolism involving the occipital cortex, visual association areas, and posterior parietotemporal region [15]. However, in a comparative study, no significant differences were observed between PDD and DLB in terms of FDG uptake while PD patients without dementia exhibited similar profiles of metabolism as healthy controls [16]. DLB is characterized by a unique pattern of occipital hypometabolism with relatively spared posterior cingulate cortex, known as the cingulate island sign because it gives the impression of the existence of an island of normal metabolism in the posterior cingulate region [17]. A study also associated the extent of cerebral hypometabolism with symptoms of visual hallucination, a higher degree of hypometabolism, mostly around visual association areas rather than in the primary visual cortex, was found in DLB patients with visual hallucination when compared to DLB patients without visual hallucinations [18]. The Dementia with Lewy Bodies (DLB) Consortium also considers the cingulate island sign (CIS) in FDG-PET as a supportive biomarker of DLB [17]. CIS shows that brain metabolism in the posterior cingulate of patients with DLB is relatively preserved compared with Alzheimer’s disease (AD) and is also known to have a negative correlation with the Braak neurofibrillary tangle stages [17]. Some studies also suggest the association between CIS and visual symptoms in DLB patients [18].

Amyloid Imaging

Pittsburgh compound B (PiB) has been extensively used as a radioligand to investigate amyloid deposition-related neurodegenerative disorders. Kantarci et al. combined antemortem positron emission tomography (PET) using PiB as a radiotracer with 39 autopsy-confirmed cases involving 14 DLB, six AD, and 19 cases of mixed pathology, which showed that a lower global cortical standardized uptake value ratio (SUVr) of PiB, successfully differentiating between cases of DLB and AD or mixed pathology with 80% sensitivity, 86% specificity, and 93% accuracy [19]. Patients with PD or PDD also have been known to show less PiB accumulation than DLB [20]. A study using tau PET 18F-flortaucipir (AV-1451) established that the significantly higher AV-1451 uptake in patients with AD when compared to the patients with probable DLB, along with medial temporal lobe uptake, was able to completely differentiate between AD and probable DLB [21]. Topographically, the AV-1451 uptake in DLB patients was localized around the posterior temporoparietal and occipital cortex [21]. Another study found the cortical [18F]AV-1451 uptake to be highly variable in DLB patients compared to the healthy variants and found the uptake to be localized in the inferior temporal gyrus and precuneus [22]. The study by Gomperts et al. also correlated clinical findings in the form of MMSE with imaging and postulated that greater AV-1451 uptake was associated with a greater degree of cognitive decline [22]. Another study also associated higher PiB retention with lower MMSE scores [23]. The findings of several molecular imaging studies have been summarized below in Table 2.

**Table 2:** Summary of molecular imaging PCA: posterior cortical atrophy; HV: healthy variants; AD: Alzheimer's disease; DLB: Dementia with Lewy bodies; PDD: Parkinson's disease with dementia; CIS: Cingulate island sign; FDG-PET: fluorine-18-fluorodeoxyglucose positron emission imaging; MMSE: Mini-Mental State Examination; MRI: magnetic resonance imaging; AV-1451: 18F-flortaucipir; PiB-PET: Pittsburgh compound B(PiB) positron emission tomography

Author	Study performed	Subject	Findings
Gupta et al.	FDG-PET	34 PCA, 38 DLB	Cerebral hypometabolism was found to be localized in the occipital cortex in DLB patients [15].
Klein et al.	FDG-PET	6 DLB, 8 PDD, 9 PD without dementia	Both DLB and PDD groups showed cerebral hypometabolism without significant differences amongst them, while PD patients without dementia didn’t show FDG binding reductions [16].
Perneczky et al.	FDG-PET	14 DLB with visual hallucination, 7 DLB without visual hallucination, 16 HV	The extent of hypometabolism in visual association areas is associated with symptoms of visual hallucination [24].
Iizuka et al.	FDG-PET, MRI with voxel-based morphometry	24 DLB, 24 AD	Higher CIS ratio in DLB compared to AD, which is associated with symptoms of visual hallucinations with CIS [18].
Kantarci et al.	PiB-PET	14 DLB, 6 AD, 19 mixed pathology of DLB and AD	Lower PiB retention in DLB patients compared to the groups with AD or mixed pathology [19].
Edison et al.	PiB-PET	13 DLB, 12 PDD, 10 PD without dementia, 41 HV	Higher PiB retention in over 80% of subjects with DLB compared to other subjects, signifying the role of amyloid-related pathology in DLB patients [20].
Kantarci et al.	AV-1451, PiB-PET	19 DLB, 19 AD, 95 HV	AD group showed higher medial temporal AV-1451 uptake than DLB. DLB group showed higher AV-1451 uptake compared to healthy variants suggesting the role of tau pathology in DLB [21].
Gomperts et al.	AV-1451, PiB-PET	7 DLB, 8 PDD, 9 PD without dementia, 29 HV	Higher AV-1451 uptake was found in the inferior temporal gyrus and precuneus in DLB and higher AV-1451 uptake was linked to lower MMSE scores [22].
Maetzler et al.	PiB-PET	9 DLB, 12 PDD, 14 PD without dementia	A greater extent of PiB binding was found to be associated with a worse MMSE score [23].

Imaging Involving Dopaminergic Activity

Many researchers agree that the evaluation of nigrostriatal degeneration is essential to distinguish between AD and DLB [25]. Dopaminergic activity can be measured with the help of dopamine transporter (DAT) imaging with SPECT using iodine 123-radiolabeled 2β-carbomethoxy-3β-(4-iodophenyl)-N-(3-fluoropropyl) nortropane or [123I]FP- CIT-SPECT as a radiotracer [25]. This showed that FP-CIT binding in the caudate and anterior and posterior putamens was significantly reduced in subjects with DLB in stark contrast to both the AD and healthy control groups [26]. A study by Walker et al. established that the sensitivity for an FP-CIT SPECT scan for DLB was 88% while specificity was 100% compared to the initial clinical diagnosis, which had a sensitivity of 75% and specificity of 42% [27]. A clinical trial involving 326 patients established that an abnormal [123I]FP- CIT-SPECT scan had a sensitivity of 77.7% for probable DLB along with a specificity of 90.4% for excluding other etiologies of dementia that are not related to Lewy body pathology [28]. DLB patients also have been known to demonstrate higher nigroputaminal fractional anisotropy (FA) values than both AD and control groups [25]. However, DAT imaging has not proved to be helpful in differentiating between DLB and Parkinson's disease with mild cognitive impairment (PD-MCI) and Parkinson's disease dementia (PDD) [29]. Another study showed that although the [123I]FP- CIT-SPECT scan is very helpful while making the distinction between AD and DLB, the specificity of the scan reduced to 67% when performing a study with patients with frontotemporal dementia (FTD) [30]. In this study, the majority of DLB patients did show notably reduced DAT binding, but a third of the FTD group also showed similar findings [30]. The findings of dopamine transporter imaging (DAT) is summarized in Table 3.

**Table 3:** Summary of findings in dopamine transporter imaging (DAT) [123I]FP- CIT-SPECT: Iodine 123-radiolabeled 2β-carbomethoxy-3β-(4-iodophenyl)-N-(3-fluoropropyl) nortropane single-photon emission tomography HV: healthy variants; AD: Alzheimer's disease; DLB: dementia with Lewy bodies; PDD: Parkinson's disease with dementia; CIS: Cingulate island sign; FA: fractional anisotropy; MMSE: Mini-Mental State Examination; MRI: magnetic resonance imaging; FTD: frontotemporal dementia

Author	Study	Subjects	Summary of findings
O’Brien et al.	[123I]FP- CIT-SPECT	33HV, 34 AD, 23 DLB, 38 PD without dementia, 36 PDD	Markedly reduced FP-CIT binding in the caudate and anterior and posterior putamen in DLB when compared to AD and HV [26].
Walker et al.	[123I]FP- CIT-SPECT	Eight DLB, nine AD, three other pathology	[123I]FP- CIT-SPECT significantly increased the diagnostic accuracy while differentiating between DLB and other pathologies with a sensitivity of 88% and specificity of 100% [27].
McKeith et al.	[123I]FP- CIT-SPECT	94 probable DLB, 57 possible DLB 147 non-DLB dementia, 28 patients without any diagnosis	Low DAT binding evaluated by [123I]FP- CIT-SPECT has a very high diagnostic correlation with a diagnosis of DLB [28].
Pardini et al.	[123I]FP- CIT-SPECT	12 HV, 18 DLB, 21 AD	Higher nigroputaminal FA values in DLB patients compared to AD and HV [25].
Chang et al.	99mTc-ethyl cysteinate dimer (ECD) SPECT	17 DLB, 16 PD, 10 HV	DLB group showed marked hypoperfusion in the frontal, parietal, thalamus, temporal regions when compared to HV [29].
Morgan et al.	[123I]FP- CIT-SPECT	12 FTD, 10 DLB, 9 AD	While SPECT scan displayed an excellent specificity of 89% in the AD group, in the FTD group the specificity reduced to 67% [30].

I-Metaiodobenzylguanidine (MIBG) Cardiac Scintigraphy

Many studies have associated Lewy body-related conditions with decreased cardiac (123) I-metaiodobenzylguanidine uptake and concluded that MIBG cardiac scintigraphy could accurately distinguish between two very common etiologies of dementia, Alzheimer’s disease and dementia with Lewy bodies [31]. (123) I-metaiodobenzylguanidine cardiac scintigraphy can reliably differentiate between two clusters of diagnosis, one involving Parkinson’s disease (PD), dementia with Lewy bodies (DLB), and rapid eye movement sleep behavior disorder (Lewy body-related conditions); and the other involving patients of AD, multiple system atrophy (MSA), progressive supranuclear palsy (PSP), vascular dementia (VaD), and frontotemporal dementia (FTD), which are essentially non-Lewy body-related disorders [31]. A study with a population of 133 subjects found that a 123I-MIBG scan had a 68.9% sensitivity and 89.2% specificity to distinguish between AD and DLB using the heart to mediastinum ratio [31]. Another study performed a three-year follow-up after making an initial diagnosis to reassess the diagnostic usefulness of 123I-MIBG scan in DLB and established a reliable correlation between abnormal cardiac sympathetic activity and a diagnosis of DLB both at early and follow-up imaging [32]. However, the presence of comorbidities like diabetes or cardiac disease can interfere with the 123I-MIBG scan and might yield false-positive results [33]. Hence, comorbidities must be strictly considered when considering the 123I-MIBG scan [33].

## Conclusions

After a thorough review, we can conclude that neuroimaging holds the key to an early and accurate diagnosis of dementia. Structural studies like CT scans help rule out various clinically relevant differentials while MRI can provide detailed volumetric assessment and identify volume loss in particular regions of the brain, which is often characteristic of a specific etiology of dementia. Both of these modalities are easier to perform, widely available, and thus very useful for the early diagnosis of dementia. Afterward, more specialized studies like SPECT and PET can help arrive at a diagnosis by differentiating between other etiologies of the similar spectrum, which are otherwise very closely related and difficult to distinguish from. This distinctly points towards a multimodal approach towards the diagnosis of DLB by integrating structural, metabolic, and molecular imaging techniques. However, further study and research are required to increase the diagnostic accuracy when differentiating between Parkinson’s disease dementia (PDD), DLB, and other disorders of a similar spectrum to facilitate the early and accurate diagnosis of DLB, leading to better patient care.
